# Biomarkers in Overactive Bladder: A New Objective and Noninvasive Tool?

**DOI:** 10.1155/2011/382431

**Published:** 2011-05-29

**Authors:** Tiago Antunes-Lopes, Sérgio Carvalho-Barros, Célia-Duarte Cruz, Francisco Cruz, Carlos Martins-Silva

**Affiliations:** ^1^Department of Urology, Hospital de S. João, 4200-319 Porto, Portugal; ^2^Faculty of Medicine, University of Porto, 4200-319 Porto, Portugal; ^3^Instituto de Biologia e Molecular e Celular (IBMC), University of Porto, 4150-180 Porto, Portugal; ^4^Institute of Histology and Embryology, Faculty of Medicine, University of Porto, 4200-319 Porto, Portugal

## Abstract

Overactive bladder syndrome (OAB) is a highly prevalent urinary dysfunction, with considerable economic and human costs. Clinical diagnosis of OAB is still based on subjective symptoms. A new accurate, objective and noninvasive test to diagnose OAB and assess therapeutic outcome is lacking. Recent studies in lower urinary tract (LUT) dysfunctions, particularly in OAB patients, indicate that urinary proteins (neurotrophins, prostaglandins, and cytokines), serum C reactive protein, and detrusor wall thickness are altered, and such changes could be used as biomarkers of the disease. Nowadays, increasing emphasis has been given to the role of urinary neurotrophins, namely nerve growth factor (NGF) and brain derived neurotrophic factor (BDNF), as key players in some urinary dysfunctions. Although recently considered to be a bladder dysfunction biomarker, urinary NGF presents low sensitivity and specificity. Preliminary results suggest that BDNF may serve as a more efficient biomarker. Even though we have to wait for future studies to confirm the potential role of NGF and BDNF as OAB biomarkers, it is already clear that neurotrophins will contribute to elucidate the physiopathological basis of OAB. Herein are reviewed the latest advances in this new and exciting field, the detection and clinical application of emerging OAB biomarkers.

## 1. Introduction

OAB is currently recognized as a chronic disorder with an overall prevalence in the adult population of above 10%, but that may exceed 40% in elderly groups [1]. According to the International Continence Society (ICS), OAB is defined as a clinical syndrome characterized by the presence of urgency, with or without urgency incontinence, usually accompanied by daytime frequency and nocturia, in the absence of proven infection or other obvious pathology [2, 3]. Urinary urgency, defined as a sudden compelling desire to void that is difficult to defer, is the unique symptom that must be present in order to establish the diagnosis of OAB [3]. However, urgency is difficult to be understood by patients and caregivers. Differentiation between urgency and urge is not always straightforward. Yet, urge is a normal bladder sensation, gradual in appearance, usually proportional to the degree of bladder filling, and that can be easily controlled by individuals. In addition, grading urinary urgency is a difficult task, which may render difficult the efficacy of a therapy. The multiple questionnaires available to quantify and grade urgency severity (USS, OABq) reflect this problem [4, 5].

One way to overcome this problem would be the introduction of an objective test for the diagnosis of OAB. During the last few years, several attempts have been made, though with limited success. Detrusor overactivity (DO) is the urodynamic hallmark of OAB. Nevertheless, this abnormality can only be identified in half of the patients, whereas normal individuals often have asymptomatic involuntary detrusor contractions [6]. In addition, urodynamics is an invasive test. These facts decrease the role of this test as a useful tool for OAB diagnosis. 

Another potential marker for OAB is detrusor wall thickness (DWT), determined by ultrasound. In patients with OAB, it has been hypothesized that frequent detrusor contractions during bladder filling result in tetanic detrusor motions and cause muscular hypertrophy. DWT is shown to be higher in OAB patients and to decrease in response to antimuscarinic treatment [7], suggesting that DWT measurement is a useful biomarker to monitor disease progression and therapeutic efficacy. However, DWT measurement may not be reproducible. Liu et al. determined DWT in normal subjects and patients with OAB dry, OAB wet and interstitial cystitis (IC). Wide variation was found among all groups. There was a trend to a higher DWT in patients with OAB, whether dry or wet, compared with normal controls and patients with IC. However, the difference was not statistical significant [7]. A recent study compared measurement of DWT by transvaginal and transabdominal ultrasound. No significant difference of transvaginal ultrasound measured DWT was noted among women with OAB dry, OAB wet, and normal controls. Inversely, transabdominally measured DWT, at bladder capacity, was significantly higher in women with OAB wet or DO [8]. Until now, studies are contradictory about the potential value of DWT as a diagnostic tool for OAB [9]. Similar problems have been detected with DWT measurement in patients with bladder outlet obstruction (BOO). Various studies showed an increase in DWT with increasing the degree of BOO and a predictive value of DWT in the diagnosis of BOO [10, 11]. In contrast, in a recent investigation, DWT was remarkably uniform whether patients had a normal urodynamic test, BOO, or DO [8]. The differences in the values of DWT obtained in various studies may be explained by the use of different ultrasound probes, with different frequencies, as well as in the resolution of ultrasound-generated images [8].

Recently, near-infrared spectroscopy (NIRS), an optical technology, has also been studied as a potential noninvasive, diagnostic tool for DO in OAB patients. NIRS detects the hemodynamic variations in tissues by the use of noninvasive measurements of changes in the concentration of tissue chromophores, such as oxyhemoglobin (O2Hb) and deoxyhemoglobin (HHb). Involuntary bladder contractions may cause changes detectable by NIRS [12]. Until now, its value to detect DO in clinical practice needs to be confirmed.

Taking into account the previous data, new simple, noninvasive tests to diagnose OAB and assess therapeutic outcome are eagerly needed. Some recent studies have focused on this new and exciting field, the detection and clinical application of OAB biomarkers. 

## 2. Neurotrophins

Nowadays, increasing attention is given to the role of neurotrophins, namely, nerve growth factor (NGF) and brain-derived neurotrophic factor (BDNF) in OAB. Neurotrophins are growth factors required by neuronal cells for differentiation, survival, and maintenance, with a broad range of activities in the central and peripheral nervous system either in the developing or in the adult mammal [13]. It has been suggested that NGF and BDNF are released from urothelial and detrusor smooth muscle cells. These neurotrophins act by binding to high-affinity receptors TrkA (for NGF) and TrkB (for BDNF), cell-surface transmembrane glycoproteins expressed in bladder urothelial cells, and primary afferents [13–15].

Low-affinity receptors, such as the p75, may also play an important role on neurotrophins effects, but this aspect is still poorly studied. 

### 2.1. Urinary Nerve Growth Factor

NGF was the first neurotrophin to be discovered, by Rita Levi-Montalcini, in the 1950s [16]. It may be synthesized by both neuronal and nonneuronal cells and plays an essential role during the development of the peripheral nervous system, regulating the survival and function of postganglionic sympathetic neurons and small-diameter primary afferents [17–20]. Upon binding to TrkA, NGF may induce the expression of several genes coding for various neurotransmitters, receptors, and voltage-gated ion channels [17]. In addition to TrkA, NGF binds to p75, a low-affinity pan-neurotrophic receptor also expressed in bladder urothelial cells and primary afferent nerves. Several clinical and experimental data have suggested an interesting link between increased levels of NGF, either in bladder tissue or urine, and DO, and OAB [21]. In animal models, NGF is released in high amounts from smooth muscle cells and urothelium of overactive bladders [14]. In addition, recent studies have revealed that acute and chronic local administration of NGF reduces bladder capacity and intercontraction interval and increases bladder reflex contractions [22–25]. Likewise, TrkA blockade or NGF sequestration decreases the high frequency of bladder contractions in animal models of bladder inflammation [26] and spinal cord transection [27, 28]. Interestingly, TRPV1 seems to be an essential downstream receptor for NGF activity in the bladder. TRPV1 knockout mice, in contrast with wild-type littermates, do not develop DO, in spite of exogenous NGF administration [26]. Also, NGF increases TRPV1 translation and activity [29]. This crosstalk between NGF and TRP family should be further investigated in the future. 

#### 2.1.1. Urinary NGF Levels in OAB Patients

Similarly to what happens in experimental studies, in humans, it has also been postulated that increased levels of NGF in urine could sensitize bladder afferent pathways and enhance bladder sensory input arriving to the central nervous system, eventually leading to DO. Supporting this hypothesis, increased levels of NGF have been found in the urine of patients with OAB, idiopathic and neurogenic DO, IC, and BOO [30–36].

Recent pilot clinical studies have shown that urinary NGF levels are significantly higher (approximately 12-fold) in patients with OAB than in normal controls [37–40]. Urinary NGF concentrations have been found to be increased in patients with OAB, particularly in those complaining of urgency urinary incontinence (OAB wet) [39, 40]. Interestingly, it has also been found that urinary NGF concentration in OAB patients seems to correlate with urgency intensity. Liu and coworkers reported on that patients classified as having modified Indevus Urgency Severity Scale (USS) scores of 3 or 4 had significantly higher NGF levels than those with a score of 2 or lower [40].

**Figure 1:**
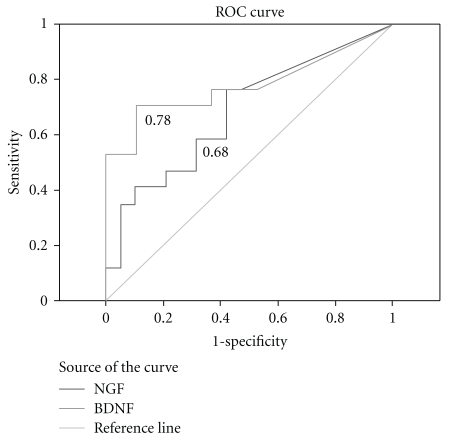
Receiver-operator characteristic (ROC) curves of urinary NGF/creatinine and urinary BDNF/creatinine in OAB patients. Notice that, for this cohort, BDNF has a better AUC than NGF.

The sensitivity and specificity was recently evaluated. Using a urinary NGF/creatinine ratio >0.05, Chen and Kuo found that the sensitivity and specificity of this test in the diagnosis of OAB was 67.9% and 93.8%, respectively [41]. In spite of being a small study, recently, Antunes-Lopes et al. found lower values of sensitivity and specificity for NGF/creatinine ratio (>200 pg/mg), with an area under the curve in receiver-operator characteristics (ROCs) analysis of 0.68 (Figure 1) [42]. In this study, there was a trend to higher NGF/creatinine ratio in OAB patients compared to healthy volunteers, but the difference did not reach statistical significance [42]. Moreover, surprisingly, in OAB patients with high urinary concentration of NGF, Birder and co-workers did not find similar increases in bladder samples obtained from the same group of patients [43]. More studies are missing to clarify this puzzling discrepancy. 

#### 2.1.2. Urinary NGF as a Marker of Response to OAB Treatment?

Antimuscarinic treatment was shown to diminish urinary NGF levels in parallel to the reduction of the USS score, with the reversal occurring upon withdrawal of the therapy [44, 45]. Also, in patients with intractable idiopathic and neurogenic DO, detrusor injection of onabotulinum toxin A has been shown to reduce urinary NGF levels [46]. According to these data, urinary NGF level could be used as a tool to monitor the therapeutic effect of antimuscarinics and detrusor BoNT-A injection in OAB. However, as these studies were not placebo controlled, some caution should be taken in the results interpretation. 

### 2.2. Urinary Brain-Derived Neurotrophic Factor

BDNF is the most abundant neurotrophin in the human body, although our knowledge about its role in normal and pathological conditions is still very limited [47]. Like NGF, BDNF also contributes to the survival and normal function of sensory neurons [48–51]. BDNF is constitutively expressed by small and medium-sized peptidergic neurons, but it is also produced by nonneuronal cells [52, 53]. Besides its well-established trophic effect on neuronal tissue and its relevance in plasticity events, the importance of BDNF in nociception has also been established [54]. BDNF is present in the spinal cord, in terminal endings of sensory fibres, colocalizing with substance P and CGRP [55]. Interestingly, its expression may be regulated by NGF [17]. 

#### 2.2.1. BDNF in Lower Urinary Tract Dysfunctions

Little is known about the role of BDNF in bladder function, both in normal and in pathological conditions, and available studies are mostly confined to experimental models of bladder dysfunction. It has been demonstrated that after chronic bladder inflammation or spinal cord injury, the synthesis of BDNF in the urinary bladder is strongly increased [56–58]. A recent study showed that BDNF sequestration improved bladder function in rats with chronic cystitis [59]. Nevertheless, BDNF sequestration did not produce any effects on bladder reflex activity of intact animals, suggesting that BDNF effect on bladder function is relevant only in pathological conditions. 

**Figure 2:**
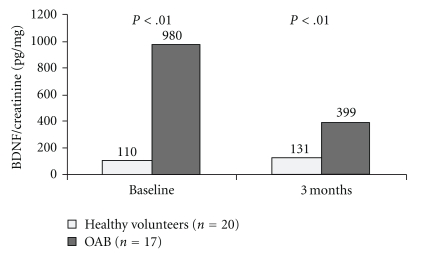
BDNF/creatinine in the urine of female healthy volunteers and OAB patients, at baseline and after 3 months of lifestyle intervention.

In a recent study, Antunes-Lopes et al. assessed urinary levels of BDNF in a population of adult healthy volunteers (20 females and 20 males) to investigate if there was a physiological pattern of secretion and if there were differences between genders. In healthy volunteers, BDNF/creatinine ratio (pg/mg) was systematically low, irrespective of gender or time of urine sampling. In contrast, urinary BDNF/creatinine ratio was significantly higher in OAB patients compared to controls (Figure 2) [60]. 

Auspiciously, the striking differences found in this preliminary observational study between OAB patients and controls suggest that urinary BDNF may serve as a potential biomarker of OAB syndrome. Using ROC analysis, the area under the curve of urinary BDNF (Figure 1) seems to support this hypothesis, but further studies, involving other centers, are necessary before a solid statement can be created [60]. 

In addition to OAB, urinary BDNF was newly evaluated in the urine of bladder pain syndrome/IC patients. The urinary concentration was high at baseline and significantly reduced after botulinum toxin administration to the bladder trigone [35]. A positive correlation could be established between BDNF decrease and LUTS improvement [35]. 

### 2.3. Neurotrophins and Intracellular Pathways: Targets for New Therapies?

The study of urinary NT in patients with OAB has provided new insights to the underlying physiopathology of this disorder. Inflammation in the urinary tract can cause an elevation of the urinary NGF level. Therefore, it can be suggested that OAB is an inflammatory disorder of the bladder [21]. NGF excretion is increased during bladder distension [36] although urinary NGF levels were augmented in patients with OAB, whether the urine was collected from an empty or a full distended bladder [61]. Although urinary NGF increases significantly in normal controls when they refer a strong desire to void NGF levels were significantly lower than in patients with OAB at first sensation of filling [61]. These results suggest that urinary NGF level increases physiologically in normal controls at strong desire to void, but raises pathologically in patients with OAB [61]. Although the precise mechanisms by which urinary NGF promotes DO and OAB are not yet defined, neurotrophins are known to influence expression and activity of receptors that modulate bladder function, like P2X3 and TRPV1 receptors. Cruz et al. showed that the latter is, in fact, essential for NGF-mediated DO [29]. This finding is important to envisage an effective strategy to counteract the consequences of high urinary NGF, levels in patients with DO [29]. In addition, neurotrophins activate intracellular signalling pathways important for micturition control, as the MAPK-ERK pathway. Some new molecules that are able to sequester NGF and other neurotrophins (e.g., BDNF) have already been tested with success in preclinical models of DO [59, 62]. In the future, it is likely that Trk antagonists or neurotrophin sequestering proteins may be eventually useful and effective treatments to control DO and OAB symptoms. 

## 3. Prostaglandins

Prostaglandins (PGs) regulate LUT function. PGs are locally synthesized in the bladder muscle and urothelium and triggered by detrusor muscle stretch, bladder nerve stimulation, bladder mucosa damage, and inflammation [63]. PGs seem to contribute to the basal ton of the detrusor and to modulate the activity of bladder nerves. PGs are involved in micturition reflex by decreasing thresholds of the stimuli necessary to trigger bladder contraction through activation of the capsaicin-sensitive afferent nerves. Therefore, PGs can be related to bladder storage symptoms in patients with OAB [63]. In an experimental study in rats, intravesical instillation of PGE2 induced detrusor contraction, while its topical application to the urethra caused urethral relaxation [64]. Activation of prostaglandin EP3 receptors exerts an excitatory effect on urinary bladder function through modulation of bladder afferent pathways [65]. 

At a clinical level, Kim et al. found that urinary levels of PGE2 and PGF2*α* in patients with OAB were significantly increased compared to a control group [63]. In addition, an inverse correlation was found between urinary PGE2 and the volume to first desire to void and the maximum cystometric capacity [63]. On the other hand, Liu et al. measured urinary levels of PGE2 in patients with OAB wet, OAB dry, IC, and controls and did not find significant differences between the subgroups [66]. 

In summary, the role of urinary PGs in the diagnosis of OAB is still controversial and needs further investigation. Moreover, nonsteroidal anti-inflammatory drugs have shown little efficacy in treating OAB. A new alternative may be the blockade of PGE receptors. Molecules like ONO-8539, a PGE2 receptor subtype EP1 antagonist, entered recently in a phase 1 study [67], but it is still too soon to make clear statements about their therapeutic potential. 

## 4. Urine Cytokines and Urine and Serum C-Reactive Protein

It has been hypothesized that OAB may be an inflammatory process of the bladder [68]. Supporting this theory, recent studies reported histological evidence of inflammation in bladder specimens from OAB patients [69, 70]. However, the biopsy-based confirmation of bladder inflammation in OAB requires an invasive and expensive procedure not exempted of morbidity. Alternatively, cytokines may represent a biomarker considering that they are elevated in biologic fluids during inflammation. In the particular case of OAB, Tyagi et al. analyzed the urine from OAB patients for selected cytokines, chemokines, growth factors, and soluble receptors. Their study revealed an elevation of several of these putative biomarkers in the urine of OAB patients (*P* < .05) [68]. Monocyte chemotactic protein-1 (MCP-1) and soluble fraction of the CD40 ligand (sCD40L) were increased more than tenfold over controls in the urine of OAB patients [68]. At least fivefold elevations were detected in the urinary levels of macrophage inflammatory protein (MIP-1*β*), IL-12p70/p40, IL-5, epidermal growth factor (EGF), and growth-related oncogene (GRO-*α*) in OAB patients compared to controls [68]. Finally, it was also noticed a threefold elevation in the urine levels of sIL-2R*α* and IL-10 in the OAB group [68].

C-reactive protein (CRP) is a widely studied general marker of inflammation and infection. Its serum levels rise dramatically during inflammatory conditions and are used to determine disease progression or treatment effectiveness. Chuang et al. undertook a study to examine CRP levels serum, urine, and bladder tissue of OAB dry and OAB wet patients [71]. Significantly higher serum CRP levels were measured in OAB patients compared to controls [71]. Interestingly, higher values were noted in OAB wet than in OAB dry [71].

In what concerns urinary levels, the same study showed that urinary CRP was barely detectable and the mRNA expression of CRP in bladder biopsies was very modest [71]. Consequently, urinary and bladder CRPs seem to be much lower than serum CRP levels, and current available methods for detecting CRP might not be sensitive enough to develop a urinary assay. On the other hand, it should be reminded that serum CRP level in patients with LUT symptoms may not specifically reflect the condition of the LUT, as its levels are influenced by any systemic inflammatory condition [71, 72]. So, at this moment, the importance of CRP as an OAB biomarker seems rather modest.
